# Color Matching of Universal Shade Resin-Based Composite with Natural Teeth and Its Stability before and after In-Office Bleaching

**DOI:** 10.1155/2022/8420890

**Published:** 2022-10-26

**Authors:** AlHanouf AlHabdan, Ahoud AlShamrani, Randa AlHumaidan, AlJohrah AlFehaid, Sara Eisa

**Affiliations:** ^1^Department of Restorative Dental Sciences, King Saud University, Riyadh, Saudi Arabia; ^2^Intern, College of Dentistry, King Saud University, Riyadh, Saudi Arabia

## Abstract

**Objectives:**

Esthetics is an essential issue for the long-term success of composite resin restoration. Therefore, this study aimed to view the esthetics of universal shade composite resin restorations and to assess its color matching before and after bleaching.

**Materials and Methods:**

Overall, 40 freshly extracted premolars were mounted in an acrylic resin mold, and Class V cavities were then prepared and restored by OMNICHROMA composite (Tokuyama Dental, Tokyo, Japan) and polished with 2-step polishing system. Baseline color analysis was performed using VITA Easyshade V digital spectrophotometer, and another color analysis was carried out 24 hours after storage in distilled water. In-office bleaching was carried out, and color measurements were taken after bleaching and 2 weeks postbleaching. The data were statistically analyzed using SPSS 26.0 Windows version statistical software. Changes were considered statistically significant at *P* = 0.05.

**Results:**

ΔE value of OMNICHROMA restoration before and after restoration was 6.474, 3.529 before and after bleaching, and 3.651 two-weeks postbleaching. In-office bleaching was effective in bleaching the OMNICHROMA specimens as the restoration showed positive ΔL^*∗*^ values, which indicated that the specimens were lighter in color after bleaching; however, the bleaching effect was not maintained after 2 weeks.

**Conclusion:**

OMNICHROMA universal shade composite resin restoration showed different color matching values with the adjacent enamel of class Vs. The material appeared lighter in shade postbleaching, and the color change was not maintained 2 weeks postbleaching.

## 1. Introduction

In the field of dentistry, resin-based composites have proven their massive popularity [1]. The outstanding evolution of resin-based composites' composition through history is undeniable, where manufacturers have highly competed to develop the best form by improving and eliminating all structural flaws that might lead to clinical difficulties [2, 3], such as structural and optical matching and blending of composite restoration within the tooth structure and the adjacent tooth. This requires the use of composite resin of different opacities and shades to match the tooth color shades, which is time-consuming for the clinician and patient [4]. Moreover, clinical effectiveness of any dental composites is dependent on their physical, chemical, and mechanical features, which are highly impacted by the oral environment and resin material properties [5].

Recently, universal composites are introduced in the market to reduce the need for a variety of composite shades in inventory, minimize the wastage of unused composite shades, minimize chair side time, eliminate the need for shade selection, and reduce reliance on shade-matching procedures. According to the developers, the main advantage of these composites relies on an enhanced color adjustment potential (CAP), defined as a “property that describes and quantifies the interaction between the physical and perceptual components of blending” [6]. These materials have a universal opacity and few VITA shades available, being recommended by developers to be used in a single shade increment that could possibly match different teeth colors [6]. Resin composite generally consists of three main components: resin matrix (organic content), fillers (inorganic part), and coupling agents [3]. The resin matrix of these composite consists mostly of Bis-GMA (bisphenol-A glycidyl dimethacrylate) mixed in different combinations with short-chain monomers such as TEGDMA (triethylene glycol-dimethacrylate), UDMA (urethane dimethacrylate), Bis-EMA (Bisphenol A polyethylene glycol diether dimethacrylate), and other monomers. The fillers are made of glass, silica, or zirconia in different filler contents and shapes [7–9].

OMNICHROMA from Tokuyama Dental Corp, Japan is one of the recent universal resin-based composites, promising clinicians' convenience in an area of a common struggle, which is shade selection. As the manufacturer states, OMNICHROMA is universal shade composite restoration with a smart chromatic technology that controls their optical properties. This method provides a perfect reflection of a specific wavelength in the tooth color [10]. Therefore, it can match all VITA classical A1-D1 shades with one universal shade. OMNICHROMA is composed of an equal size of zirconium dioxide (ZrO_2_) mixed with supra-nanospherical filler of silicon dioxide (SiO_2_) with a 260 nm particle size in addition to a round-shaped composite filler, which has the same qualities. According to the manufacturer, OMNICHROMA becomes more translucent after polymerization with a refractive index of 1.47 before and 1.52 after polymerization. This is according to previous research that detected a strong interconnection between the translucency parameter and the blending effect that is related to color shifting [10]. This shadeless composite once placed in the cavity preparation will immediately take the color of the underlying and surrounding dentin and enamel, saving both the clinician and patient's time and eliminating the shade selection step.

Hydrogen peroxide (HP), with its various concentrations from 3% to 40%, decomposed into hydroxy-free radicals under light or heat irritation and is the widely used bleaching agent to dissociate double bonds or ring structures present within stains [11–14]. The bleaching effectiveness is mainly related to the bleaching protocol used including its HP concentration, bleaching time, and the composition of restorative materials, such as the structure of the resin matrix in addition to the properties of the filler particles [15–17]. Dental bleaching can be performed on both vital and nonvital teeth [14]. Mainly, two techniques are considered for dental bleaching professional or at home. Professional treatment is performed by the dentist; while home treatment can be done at home by the patient [14]. Although the results of HP on the color change of resin-based composites are still controversial, it is mainly agreed that different types of resin-based composites reveal different resistance to bleaching [18–20]. Bleaching materials eliminate the extrinsic stains, but they do not bleach the composite as they used to bleach tooth structure. Thus, once the bleaching agent is applied, the color of the composite resin-based restoration may not always be the same as that of the adjacent bleached tooth structure [21].

Due to the limited research upon universal resin-based composite, this research aims to evaluate OMNICHROMA composite color matching after in-office bleaching with the tooth structure and its color stability pre- and post-treatment.

## 2. Materials and Methods

### 2.1. Specimen Preparation

This study was approved by the institutional review board of King Saud University project No (E-20-4918) and College of Dentistry Research Center of King Saud University No (IR 0353).

Twenty freshly extracted premolars were collected, washed, and stored in dark glass containers in 1% (v/v) thymol solution at 4°C after extraction until use. Samples were checked carefully to select teeth that are caries-, cracks-, fluorosis-, and restoration-free. IsoMet 2000 Precision Saw (Buechler Ltd, Lake Bluff, IL, USA) was used to cut the roots below the furcation. Sectioned samples were then mounted in an acrylic resin mold where the sectioned surface was positioned facing the resin. Samples were numbered and the color of each specimen where class V will be placed was recorded using a VITA Easyshade V digital spectrophotometer (VITA Zahnfabrik, Bad Säckingen, Germany). The Class V cavity outline (5 mm in width and height) was drawn with an indelible pencil and located at the junction of the middle and gingival third of each specimen. The cavities were then prepared using 330 carbide burs following the outline to a 2 mm in depth. The bur was discarded after 5 cavities. All cavities were etched using 37% phosphoric acid for 15 seconds and then washed and gently air dried. One-component self-etch light-cured adhesive (Bond Force, Tokuyama Dental, Tokyo, Japan) was applied to the cavities according to the manufacturer's instruction using microbrush, air-thinned, and light cured. The cavities were then filled with OMNICHROMA composite (Tokuyama Dental, Tokyo, Japan) using a composite gun and a regular plastic instrument and then light cured for 40 seconds using a calibrated LED light-curing device (Bluephase G2; Ivoclar Vivadent, Schaan, Liechtenstein). The restorations were then finished and polished with 2-step polishing system Enhance PoGo disks (Dentsply Caulk, Milford, DE, USA). The specimens were stored in distilled water at 37°C for 24 hours, after which the shade of the restoration was recorded. In-office bleaching was carried out using 40% HP (opalescence Boost, Ultradent Products, Inc.; USA) according to manufacturer's instructions for 20 mins, three consecutive times. Color measurements were performed immediately after bleaching and 2 weeks postbleaching. The measurement was taken three times for each tooth, and the average of these measurements was considered the measured data.

### 2.2. Color Analysis

**Figure 1:**
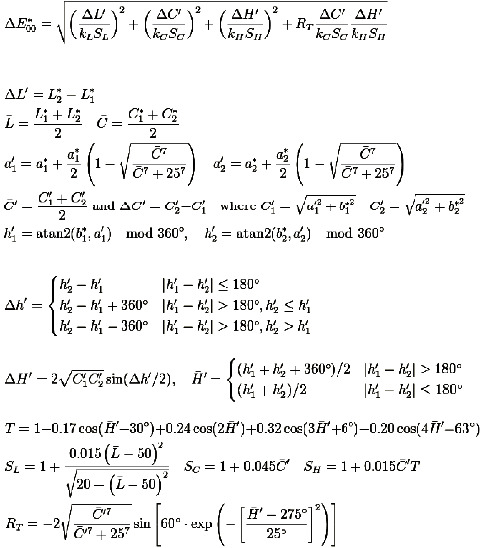
CIEDE2000 (ΔE_00_) color difference formula used in this study.

Color analysis was carried out using VITA Easyshade V digital spectrophotometer (VITA Zahnfabrik, Bad Säckingen, Germany), immediately, 24 hours after storage in distilled water, immediately after bleaching, and 2 weeks postbleaching. The device was calibrated and used according to manufacturer's instruction. Color measurements were recorded after the probe tip of the Easyshade placed perpendicular in contact with the center of class V area and restoration. L^*∗*^a^*∗*^b^*∗*^ coordinates of the CIE system (the Commission Internationale de l'Eclairage) of each specimen were recorded at tooth structure prepreparation, 24 hours after restoration, immediately after bleaching, and 2 weeks postbleaching. While L^*∗*^ coordinate represents color lightness, varying from white to black, and a^*∗*^ and b^*∗*^ coordinates represent the chroma of the color with the axes ranging from green to red and blue to yellow, respectively. C^*∗*^ is another value that is called metric chroma and it is given by equation C^*∗*^ = (*a*^2^ + *b*^2^)^1/2^. ΔE_00_ values were then calculated at four stages of the experiment: at tooth structure prepreparation, after restoration placement, immediately postbleaching, and 2 weeks postbleaching using the following formula shown in Figure 1 [22]:

### 2.3. Data Analysis

Data were analyzed using SPSS 26.0 statistical software to calculate the means and standard deviations for the L^*∗*^a^*∗*^b^*∗*^ coordinates of each group. The two-tailed *t*-test was used to compare the mean values of L^*∗*^, a^*∗*^, b^*∗*^, and ΔE values at experiment stages. A *P*-value of ≤0.05 was used to report the statistical significance of results.

## 3. Results

Descriptive statistics (mean and standard deviation) of L^*∗*^, a^*∗*^, and b^*∗*^ values at prepreparation, immediately after restoration with OMNICHROMA, postbleaching, and 2 weeks postbleaching are shown in Table 1. The highest L^*∗*^ (87.330 ((4.428)) values were obtained immediately after bleaching measurement, whereas the lowest value (73.685 ((9.059)) was associated with prerestoration measurement. High L^*∗*^ values indicate the specimen became lighter, whereas lower L^*∗*^ values indicate that the specimen became darker. Regarding a^*∗*^ and b^*∗*^ values, the highest was obtained from prerestoration measurement (2.761 ± (1.590)), 35.464 ± (4.279)), which indicated that the specimen shifted in color toward red and yellow, respectively. In addition, the lowest a^*∗*^ and b^*∗*^ values were associated with after restoration (0.704 ± (0.568)) and 2 weeks postbleaching measurements (20.795 ± (3.286)), which indicates the specimen color shifted toward green and blue, respectively.

Table 2 presents the color difference values (ΔE_00_) between tooth structure and after restoration (6.474) where all the values of L^*∗*^, a^*∗*^, and b^*∗*^ were significantly statistically different (*P* < 0.000) before and after restoration. ΔL^*∗*^ were positive values indicating an increase in the lightness of the restoration, whereas Δa^*∗*^ and Δb^*∗*^ were negative values, indicating shifting of color toward the green and blue values, respectively.

ΔE_00_ values after bleaching and two-weeks after bleaching were 3.529 and 3.651, respectively (Tables 3 and 4) with no statistically significant difference (*P* > 0.05) found between them (Table 5). ΔL^*∗*^ values only have found to be significantly statistically different (*P* < 0.000) at the two treatment stages (after bleaching and two-weeks after bleaching).

## 4. Discussion

Color matching is important for the success of resin composite restoration. It is considered as one of the most important physical features of composite material. Color changes can occur owing to a variety of etiologic variables, which include either or both extrinsic or intrinsic factors [5]. Intrinsic discoloration may arise as a result of a physicomechanical reaction within the material. Extrinsic discoloration is defined as staining in the superficial layer of a resin composite. They occur as a result of water sorption, smoking, and nutrition habits [23]. The present study was attempted to determine the total color matching of teeth restored with OMNICHROMA universal shade composite resin restoration with the surrounding tooth structure. The literature on this novel universal shade resin-based restorative material (OMNICHROMA) is sparse and insufficient, justifying the need for further studies [6, 24, 25].

CIE Lab color system has been used in this study to identify the color measurement of OMNICHROMA resin-based composite using a VITA Easyshade spectrophotometer. The CIE Lab color system is known for its international validity, ease of use, and superior reliability. It describes the color in three coordinates, L^*∗*^, which represents color lightness, a^*∗*^, which represents the chroma in red-green direction, and b^*∗*^, which represents chroma in yellow-blue direction. These coordinates are calculated manually or through computer program through the aforementioned formula to produce ΔE^*∗*^ values. Various formulae are used to determine the difference between two colors. The classical Euclidean formula (ΔE^*∗*^ab) and the recently introduced CIEDE2000 (ΔE_00_) formula are the most commonly used nowadays [26]. Although, the ΔE^*∗*^ab formula has undergone changes over the time. It does not provide information about the two variables that differ the most, which are the direction and magnitude of the colorimetric variables [26]. In 2001, the International Commission on Illumination (CIE) recommended its most recent discovered color difference formula, CIEDE2000 (ΔE_00_), which is considered the ISO/CIE (ISO IOS-J03) standard [27]. Most researchers agree that the CIEDE2000 formula better reflects the color differences perceived by the human eye than the classical CIELAB formula [26, 28–30]. When ΔE values are detected to be 0, it indicates that there is no change in color between the compared samples. If ΔE was between 0 and 3.2, the change in color is present; however, this change is undetectable visually and might be clinically acceptable. Moreover, if ΔE is 3.3 and above, the change is detectable visually and might be considered unacceptable [30–32].

As per manufacturer, OMNICHROMA does not include pigments, and its color properties are based on the color of the surrounding structure and it is based on a smart chromatic technology with the goal of controlling the optical properties of the resin composite. However, in our study, there was a statistically significant difference between OMNICHROMA universal shade composite resin restoration and the tooth structure around it in the L^*∗*^ and b^*∗*^ coordinates as shown in Table 1. This result was in agreement with a recent study by Alhamdan et al who found that color matching was better in the conventional resin-based composite than in the universal shade composite. In addition, they discovered that different tooth shades had an effect on the color matching ability of the universal shade resin-based composite [33]. The review of the overall differences in color coordinates recorded during this study between the intact teeth and the restored teeth with OMNICHROMA indicates that the difference in the lightness coordinate ΔL had a positive value for all teeth, which means that there was a shift of the restoration color to a lighter shade. Regarding Δa^*∗*^, the difference was with a negative value, which indicates shifting of the restoration color to the green scale. However, this difference was not statistically significant. Additionally, the difference in the blue-yellow coordinate Δb^*∗*^ was statistically significant with a negative value, which indicates the restoration color shifting to the blue scale. Contradictory results were obtained by Mohamed et al [34], Mourouzis et al. [35], and Gamal et al. [36] where they found a close match between the OMNICHROMA universal shade composite resin restoration and the surrounding tooth structure among all three coordinates.

The bleaching technique employed in this study is a simulation of in-office bleaching using 40% HP concentration, which is a chemically activated system. ΔE^*∗*^ values of OMNICHROMA restoration before and after bleaching were 3.529 and 3.651 two weeks postbleaching with no statistically significant difference. These recorded values are considered higher than the perceivable threshold of 3.3, which means that there was a noticeable color change. This result was in agreement with Evans et al. [37] who demonstrated the ability of OMNICHROMA to change shade after surrounding tooth structure was bleached. The values of L^*∗*^ indicate that in-office bleaching was able to bleach OMNICHROMA specimens as the restoration shows positive ΔL^*∗*^ values, which indicate that the specimens were lighter in color after bleaching.

Universal shade resin-based composites require more studies to evaluate and assess other physical and optical properties of the material, such as translucency, opacity, and surface roughness. Additionally, as for other *in vitro* studies, there are several limitations to our study, which include studying the effect of oral cavity conditions, aging and finishing and polishing protocols on the shade matching ability, and the color stability of the material.

## 5. Conclusions

Within the limitations of this study, it can be concluded that OMNICHROMA universal shade composite resin restoration showed different color matching values with the adjacent enamel of class Vs as ΔE is higher than the perceivable values. After bleaching was performed, OMNICHROMA universal shade composite resin restoration showed the ability to appear lighter in shade as the surrounded enamel becomes brighter. However, this color change was not stable when recorded 2 weeks postbleaching.

## Figures and Tables

**Table 1:** Descriptive statistics of L^*∗*^, a^*∗*^, and b^*∗*^ at prerestoration, after restoration, postbleaching, and 2 weeks postbleaching.

Experiment stage	L^*∗*^	a^*∗*^	b^*∗*^
	Mean ± (SD)		
Prerestoration	73.685 ± (9.059)	2.761 ± (1.590)	35.464 ± (4.279)
After restoration	82.057 ± (4.768)	0.704 ± (0.568)	23.285 ± (2.879)
Postbleaching	87.330 ± (4.428)	0.864 ± (.681)	22.392 ± (2.845)
2 weeks postbleaching	81.954 ± (6.060)	0.902 ± (.603)	20.795 ± (3.286)

**Table 2:** Color difference in L^*∗*^, a^*∗*^, and b^*∗*^ at tooth structure and restoration measurements.

Treatment stage	L^*∗*^	a^*∗*^	b^*∗*^
	Mean ± (SD)		
Prerestoration	73.685 ± (9.059)	2.761 ± (1.590)	35.464 ± (4.279)	
Restoration	82.057 ± (4.768)	0.704 ± (0.568)	23.285 ± (2.879)	ΔE_00_
Δ	8.372 ± (4.291)	−2.057 ± (1.022)	−12.179 ± (1.4)	6.474
*P*-value	0.000^*∗*^	0.000^*∗*^	0.000^*∗*^	

^*∗*^indicates a significant difference between tooth structure and restoration values.

**Table 3:** Color difference in L^*∗*^, a^*∗*^, and b^*∗*^ at restoration and after bleaching measurements.

Treatment stage	L^*∗*^	a^*∗*^	b^*∗*^
	Mean ± (SD)		
Restoration	82.057 ± (4.768)	0.704 ± (0.568)	23.285 ± (2.879)	
Postbleaching	87.330 ± (4.428)	0.864 ± (0.681)	22.392 ± (2.845)	ΔE_00_
Δ	5.273 ± (0.34)	0.16 ± (0.113)	−0.893 ± (0.034)	3.529
*P*-value	0.000^*∗*^	0.257	0.166	

^*∗*^indicates a significant difference between restoration and postbleaching values.

**Table 4:** Color difference in L^*∗*^, a^*∗*^, and b^*∗*^ at after bleaching and 2 weeks after bleaching measurements.

Treatment stage	L^*∗*^	a^*∗*^	b^*∗*^
	Mean ± (SD)		
Postbleaching	87.330 ± (4.428)	0.864 ± (0.681)	22.392 ± (2.845)	
2 weeks postbleaching	81.954 ± (6.060)	0.902 ± (0.603)	20.795 ± (3.286)	ΔE_00_
Δ	−5.376 ± (1.632)	0.038 ± (0.078)	−1.597 ± (0.441)	3.651
*P*-value	0.000^*∗*^	0.792	0.022	

^*∗*^indicates a significant difference between postbleaching and 2 weeks postbleaching values.

**Table 5:** Color difference recorded in L^*∗*^, a^*∗*^, and b^*∗*^ values between after restoration and after bleaching and between after bleaching and 2 weeks after bleaching in addition to ΔE values.

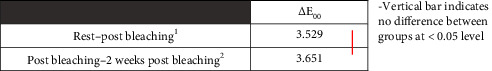

## Data Availability

Data are available upon reasonable request to the corresponding author.
